# Clinical and pelvic floor ultrasound characteristics of pelvic organ prolapse recurrence after transvaginal mesh pelvic reconstruction

**DOI:** 10.1186/s12905-022-01686-1

**Published:** 2022-04-05

**Authors:** Zhenzhen Liu, Gaowa Sharen, Pan Wang, Liyuan Chen, Li Tan

**Affiliations:** 1 Department of Ultrasound Medicine, Peking Union Medical College Hospital, Chinese Academy of Medical Sciences/Peking Union Medical College, Beijing, China; 2 Department of Health Management, Peking Union Medical College Hospital, Chinese Academy of Medical Sciences/Peking Union Medical College, Beijing, China; 3 Department of Ultrasound, The Sixth Hospital of Beijing, Beijing, China; 4 Department of Ultrasound, Bozhou People’s Hospital Affiliated to Anhui University of Technology, Anhui Province, China

**Keywords:** Ultrasound, Pelvic organ prolapse, Recurrence, Surgery, Risk factor, Levator ani hiatus

## Abstract

**Background:**

Recurrence of pelvic organ prolapse (POP) after transvaginal mesh (TVM) implantation pelvic floor reconstruction surgery remains an unresolved problem in clinical practice. In this retrospective observational study, clinical and pelvic floor ultrasound (PFUS) parameters were analyzed in order to identify high-risk factors of POP recurrence.

**Methods:**

The clinical and PFUS data from September 2013 to November 2019 of patients who underwent TVM were retrospectively analyzed. The patients with prolapse recurrence on postoperative follow-up diagnosed by PFUS were selected as case group, the clinical and PFUS parameters of them were compared with the control group in which the patients had no sign of prolapse recurrence. Univariate and multivariate regression analyses were performed based on age, BMI, gravidity, parity, surgical history (non-POP hysterectomy and incontinence-or-POP surgery), preoperative POP stage, follow-up in years, levator avulsion and hiatal area (HA) on Valsalva.

**Results:**

Altogether 102 patients entered the study and the median interval between PFUS and TVM surgery was 2.5 years. Univariate analysis showed that levator avulsion and HA were significantly different between case group and control; multivariate regression analysis showed that only HA was related to prolapse recurrence after TVM (OR = 1.202, 95% CI 1.100–1.313, *P* < 0.001). The area under the ROC curve was 0.775 (95% *CI* 0.684–0.867, *P* < 0.001).

**Conclusions:**

Hiatal area on Valsalva was related to prolapse recurrence after TVM surgery and it is an important parameter for postoperative follow-up of TVM surgery.

## Background

Transvaginal mesh (TVM) pelvic floor reconstruction is an effective treatment method for moderate to severe pelvic organ prolapse (POP) in middle-aged and elder women [1]. Compared with traditional native-tissue repair, TVM surgery can achieve better anatomical repositioning, improve patients’ bulge symptoms, and reduce the recurrence rate of anterior vaginal wall prolapse [2, 3]. However, recurrence after TVM surgery remains a significant problem in clinical practice. A high-quality randomized controlled trial from 2016 has shown that the recurrence rate of prolapse after TVM surgery may vary from 0 to 18% [2]. Recurrence was correlated with the difference in surgical techniques and individual differences among patients. Moreover, a meta-analysis involving 25 studies and 5082 patients who underwent prolapse surgeries (with or without mesh) suggested that major risk factors for recurrence are levator avulsion, preoperative prolapse stage 3–4, family history, and hiatal area (HA) [4].

Diagnostic methods for POP prolapse recurrence include clinical examination, questionnaires, and imaging [5, 6]. The International Continence Society Pelvic Organ Prolapse Quantifications System (ICS POP-Q system) has some unresolved problems, including the inability to accurately identify levator coactivation, insufficient duration of Valsalva maneuver, and insufficient quality control. With reference to imaging techniques, 3D/4D pelvic floor ultrasound (PFUS) is among the most common non-invasive imaging tools for patients with POP [7]. It can help to make better differentiation diagnosis of different forms of pelvic organ prolapses, and eliminate the above-mentioned defects of ICS POP-Q system. Previous studies on TVM surgery of China have been mainly focused on the assessment of surgical efficacy (POP-Q score, questionnaire assessment, imaging, etc.) [8–10], diagnosis and treatment of surgical complications (mesh exposure and erosion, etc.) [8–10], and the effect of surgery on sexual function [3]; yet, studies on postoperative recurrence in Chinese patients undergoing PFUS have been rarely reported.

In this study, PFUS was used in Chinese patients who underwent TVM pelvic floor reconstruction surgery to diagnose prolapse recurrence. The mesh position and movement during Valsalva maneuver was recorded. The support failure types of anterior compartment mesh were reported. Clinical and PFUS parameters were compared between case and control group to find high-risk factors for POP recurrence.

## Methods

This study was approved by the Ethics Committee of Peking Union Medical College Hospital of Chinese Academy of Medical Sciences.

### Study settings

This was a cross-sectional study. The clinical and PFUS volume data of patients who underwent TVM pelvic floor reconstruction surgery and were followed up with PFUS from September 2013 to November 2019 in our hospital were retrospectively analyzed. Our hospital is one of the largest diagnosis and treatment centers for pelvic floor disorders in China. All TVM surgeries were performed by the same doctors team. Inclusion criteria were the following: patients who underwent TVM surgery because of POP in our hospital; the patient had clinical review data as well as PFUS data; the interval between PFUS and surgery was at least 10 months. All patients were grouped into case and control group according to post operation PFUS. The patients with prolapse recurrence in any compartment diagnosed by PFUS were grouped to case group. The patients without any of the above abnormality constituted the control group. The general data and clinical and ultrasound parameters between the case group and the control group were compared in order to identify the risk factors for POP recurrence after TVM.

The patients’ inpatient medical records were collected, including patient age, gravidity, parity, history of gynecologically-related surgery (1-hysterectomy for non-prolapse causes; 2-surgery of POP or incontinence causes), preoperative prolapse ICS POP-Q stage (ICS POP-Q stage 3–4 patients were classified as severe POP group, ICS POP-Q stage 2 or lower patients were classified as mild POP group), and date of surgery. The body mass index (BMI) of the patients at the date of PFUS examination were recorded. Follow-up in years was calculated as the interval between surgery and first ultrasound showing prolapse recurrence (cases) and the interval between surgery and last scan (controls).

GE Voluson E8 or E10 diagnostic ultrasound machine (GE Company) with transabdominal 4D probes were used. The PFUS of all patients were finished by one doctor (L. Tan). With bladder lithotomy position, the patients were asked to perform the maximum Valsalva maneuver (forced expiration against a closed glottis and contracted diaphragm and abdominal wall, lasting at least 6 s). The midsagittal view was taken to observe the descent of pelvic structures including the bladder, vault of vagina, bowels, and rectum. A horizontal reference line (H line) that passed the posteroinferior margin of the symphysis pubis was used as the reference line. In addition, three-dimensional volumetric imaging was performed to observe the integrity of the levator ani in the TUI model during maximal pelvic floor contraction; the HA was measured during the maximum Valsalva maneuver. The mesh-anchor failure type of cystocele cases was reviewed by two doctors (Z. Liu and L. Chen), and were classified into anterior failure, apical failure and global failure according to reference [11].

The diagnostic criteria for significant ultrasound prolapse were [12]: cystocele (the lowest point of posterior wall of the bladder is located 10 mm or more below the reference line), enterocele (the lowest point of bowel reached the reference line or lower) and true rectocele (rectal ampulla bulged ≥ 15 mm in depth toward the posterior vaginal wall in acute angle). POP recurrence was diagnosed when cystocele, enterocele or rectocele (at least one of the three aspects) was detected on ultrasound. Levator-urethra gap (LUG) 25 mm was taken as cut-off value to diagnose the levator avulsion.

### Statistical analysis

SPSS 26.0 software was used; measurement data conforming to normal distribution were expressed as mean ± standard deviation, and those not conforming to normal distribution were expressed as range, median, 25% and 75% quartiles. Age, BMI, gravidity, parity, surgical history, preoperative prolapse ICS POP-Q stage, follow-up in years, levator avulsion, and HA of case and control groups were compared using t-test (comparison of continuous variables conforming to normal distribution), U test (comparison of continuous variables not conforming to normal distribution) and chi-square test (comparison of categorical variables). Afterward, variables with *P* < 0.10 were entered into Logistic regression analysis. ROC curve was plotted and the area under the curve (AUC) was calculated. *P* < 0.05 (two-tailed) was considered to be statistically significant.

## Results

Altogether 102 patients entered our analysis. The patients mean age was 65 years (53–77). Mean parity was 2 (0–5). Mean BMI when they came for review was 24.49 (18.82–30.08). 10 of them had a history of non-POP hysterectomy; 15 had surgery history of POP or incontinence. Concomitant hysterectomy was performed in all of the remaining patients. 10 patients received concomitant incontinence surgery (mid urethral tape surgery). The median interval between PFUS and TVM surgery was 2.5 years (0.8–10.8 years).

**Fig. 1:**
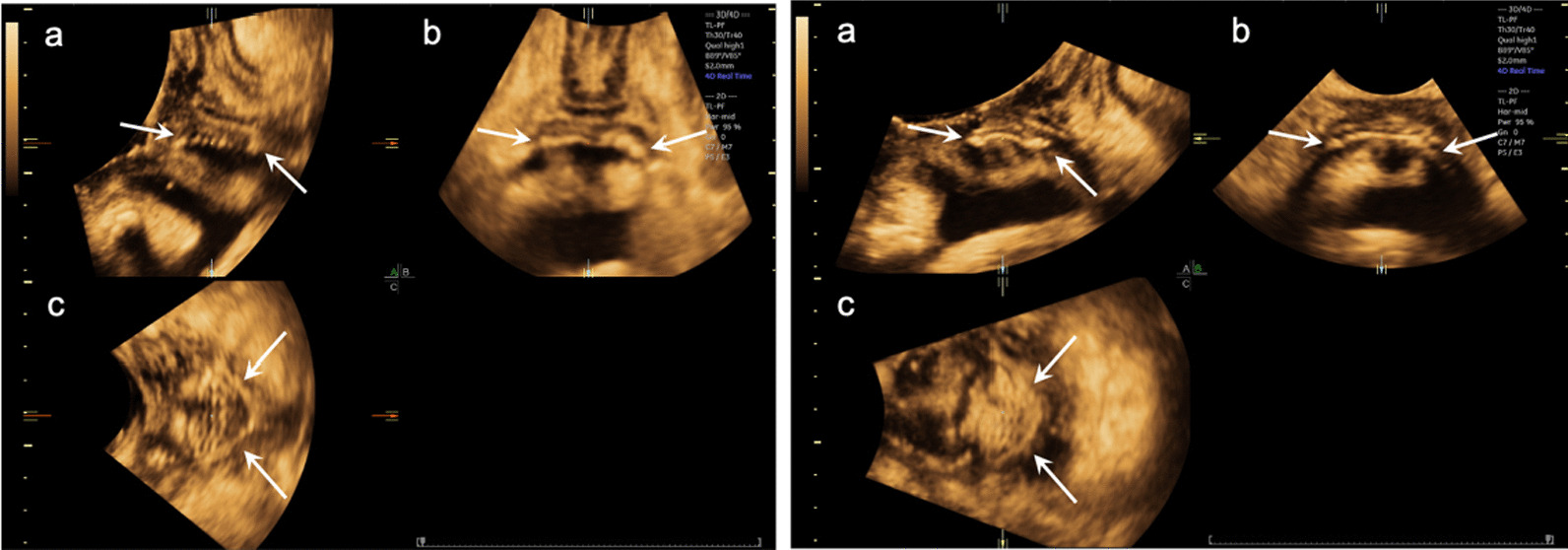
An example of mesh image in a patient under rest and Valsalva Maneuver. Anterior compartment mesh (arrows) visible in the mid-sagittal (**a**), coronal (**b**) and axial (**c**) planes in a patient under rest phase (left-side) and maximum Valsalva maneuver (right-side)

On PFUS, cystocele was diagnosed in 37 patients, among whom 6 were in combination with enterocele or rectocele; Enterocele, rectocele, or both but without cystocele were diagnosed in 17 patients. Altogether 54 patients composed the case group. Anterior vaginal wall meshes could in all cases be observed, and mesh movement was recorded on Valsalva Maneuver (Fig. 1). There were altogether 7 cases in which the meshes were suspected to be contracted, 4 in case group and 3 in control group. Among the 37 patients of cystocele recurrence, anterior failure was noted in 11 (29.7%), apical failure in 7 (18.9%) and global failure in 15 (40.54%) patients.

The distribution and comparison of general data, clinical characteristics, and ultrasound features of patients in the case group, control group and total patients were shown in Table 1. Only levator avulsion (27.8% vs. 4.2%, *P* = 0.001) and HA (26.35 cm^2^ vs. 21.70 cm^2^, *P* < 0.001) were significantly different between the two groups.

**Table 1:** Clinical and ultrasound parameters comparison between the recurrence (patients with cystocele, enterocele or rectocele) and no recurrence groups after transvaginal mesh surgery

Parameters	Recurrence		No recurrence		Total patients		Statistics	
	Value	Number	Value	Number	Value	Number	Statistics value	*P* value
General data								
Age (mean ± SD)	64.50 ± 4.91	54	65.90 ± 5.17	48	65.16 ± 5.05	102	1.399	0.165
BMI at the time of clinical review (mean ± SD)	25.00 ± 2.65	33	24.01 ± 2.40	35	18.82–30.08, 24.49 ± 2.55	68	− 1.603	0.114
Gravidity (range, median (25%, 75% quartiles)	1–6, 3 (2, 4)	52	0–7, 3 (2, 4)	45	0–7, 3 (2, 4)	97	3.048	0.880
Parity (range, median (25%, 75% quartiles)	1–5, 1.5 (1,2)	52	0–5, 2 (1, 2)	45	0–5, 2 (1, 2)	97	5.624	0.345
Clinical parameters								
Previous hysterectomy for non-prolapse factors, number (%)	6 (11.1)	54	4 (8.3)	48	10 (9.8)	102	0.222	0.638
Previous incontinence or prolapse surgery, number (%)	11 (20.4)	54	4 (8.3)	48	15 (14.7)	102	2.935	0.087
3–4° anterior prolapse, number (%)	52 (96.3)	54	48 (100)	48	100 (98.0)	102	1.813	0.178
3–4° central prolapse, number (%)	42 (77.8)	54	32 (66.7)	48	74 (72.5)	102	1.575	0.209
3–4° posterior prolapse, number (%)	8 (14.8)	54	7 (14.6)	48	15 (14.7)	102	0.001	0.974
Follow-up in years (range, median (25%, 75% quartiles)	0.9–10.8, 2.75 (1.275, 4.450)	54	0.8–7.1, 2.30 (1.325, 3.350)	48	0.8–10.8, 2.50 (1.30, 3.575)	102	1397.500	0.496
Ultrasound features								
Levator avulsion, number (%)*	15 (27.8)	54	2 (4.2)	48	17 (23.6)	72	10.200	0.001
Hiatal area on Valsalva (range, median (25%, 75% quartiles)*	17.4–50.4, 26.35 (23.85, 31.30)	54	11.7–36.3, 21.70 (17.00, 24.90)	47	11.7–50.4, 24.40 (21.65, 29.35)	101	1967.500	0.000

*Parameter showed significant difference between case and control groups (*P* < 0.05)

**Fig. 2:**
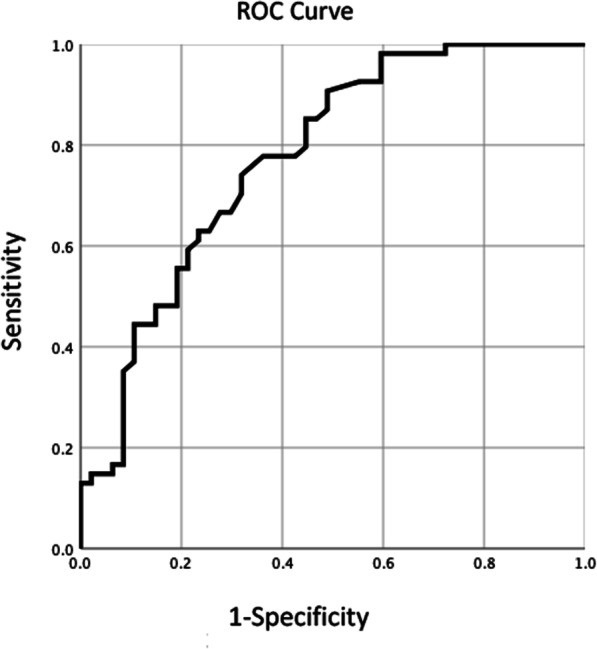
The ROC curve for the hiatal area in prolapse recurrence

Levator avulsion, HA and POP or incontinence surgery history entered into Logistic regression analysis (*P* = 0.001, < 0.001 and 0.087, respectively). The analysis results showed that only HA was related to recurrence of POP after TVM, OR = 1.202, 95% *CI* 1.100–1.313 (*P* < 0.001). The ROC curve for HA in prolapse recurrence diagnosis was shown in Fig. 2, with AUC 0.775 (95% *CI* 0.684–0.867, *P* < 0.001).

## Discussion

In this cross-sectional study, we analyzed a number of clinical and ultrasound parameters to find high-risk factor(s) of prolapse recurrence after TVM surgery. The parameters included age, BMI at the time of clinical review, gravidity, parity, previous surgical history, preoperative prolapse stage, follow-up in years, levator avulsion, and HA. The prolapse recurrence in our study was diagnosed by ultrasound as cystocele, enterocele or rectocele (at least one of the three). Altogether 54 patients of case group and 48 patients of control group entered our study. The multivariate regression analysis showed that only HA was related to prolapse recurrence with an OR 1.202 (95% *CI* 1.100–1.313), which means that for every 1 cm^2^ increase in HA, the probability of recurrence of prolapse after TVM increased by 20%. The prediction of HA for prolapse recurrence showed an AUC of 0.775 (95% *CI* 0.684–0.867).

Previous studies have suggested that levator avulsion is also an independent risk factor for prolapse and recurrence after prolapse surgery [5, 13, 14]. An observational study of Dietz et al. [6] published in 2014 that included 4 centers and 334 patients who underwent conventional ± mesh surgery suggested that the use of mesh, levator avulsion, and HA were independent risk/protective factors for POP recurrence in both clinical and ultrasound diagnosis, with ORs of 0.41, 1.93, and 1.04, respectively. Compared with this study, the possible reasons of the different result of our study maybe the following aspects: First, our study population was different, with all patients included in our study were Chinese, and underwent mesh implantations, and with a different follow-up time range (0.8–10.8 years (median: 2.5) of ours vs. 0.26–6.39 years (mean: 2.51) of Dietz et al.) Second, while the distribution of avulsion and HA both showed differences between the case and control groups in univariate analysis, only hiatal area remained significant on multivariate analysis. This may be due to power issues or due to the association of the two factors, as avulsion directly causes HA enlargement.

Our study has a few limitations. As a cross-sectional study, HA and levator avulsion were both observed after surgery and at the same time of prolapse diagnosis. So, the cause-and-effect relationship between clinical or ultrasound parameters and clinical prognosis maybe confused. At this point, our interpretation is that it appears unlikely that either avulsion or hiatal ballooning would be greatly changed by surgery. And one previous study has showed that hiatus area enlargement was the cause but not effect of prolapse and its recurrence after prolapse surgery [15]. Another study on the avulsion diagnosis pre- and postoperatively demonstrated highly consistent of this parameter at the two time-points and so the postoperative diagnosis of avulsion could be used as a predictor of prolapse recurrence [16]. Besides, this was a retrospective study with a small sample size; some data, such as BMI at the time of clinical review, were incomplete; patients’ family history of POP, postoperative clinical POP-Q stage, and postoperative questionnaire assessment were not included in this study. Further study is necessary.

Our study focused on ultrasound parameters in predicting prolapse recurrence. For clinicians who do not have access to imaging, “Gh (anterior–posterior diameter of genital hiatus) + Pb (anterior–posterior diameter of perineal body)” on the ICS POP-Q if also measured on Valsalva may act as a surrogate for HA. Previous studies have shown the correlation between value of (Gh + Pb) in POP-Q scoring and HA in PFUS [17, 18]. And what’s more, “Gh” has been shown to be an independent predictor of prolapse recurrence [19].

## Conclusion

In this retrospective study, hiatus area under Valsalva was related to prolapse recurrence after TVM surgery at a median follow-up length of 2.5 years. Ultrasound could help clinicians focus on patients who are at high risk of prolapse recurrence. Clinical physicians should pay more attention to patients with Hiatus area enlargement and actively take interventions in order to achieve better prognosis of these patients.

## Data Availability

The datasets used and/or analyzed during the current study are available from the corresponding author on reasonable request.

## Abbreviations

POP: Pelvic organ prolapses
TVM: Transvaginal mesh
PFUS: Pelvic floor ultrasound
HA: Hiatal area
ICS POP-Q system: The International Continence Society Pelvic Organ Prolapse Quantifications System
BMI: Body mass index
AUC: The area under the curve
Gh: Genital hiatus
Pb: Perineal body
